# Synchronous primary neuroendocrine and mucinous epithelial tumors present in the same appendix. Case report of 2 patients

**DOI:** 10.1016/j.ijscr.2020.01.022

**Published:** 2020-01-26

**Authors:** Paul H. Sugarbaker, Almog Ben-Yaacov, David Hazzan, Aviram Nissan

**Affiliations:** a Program in Peritoneal Surface Malignancies, MedStar Washington Hospital Center, Washington, DC, USA; b Department of General and Oncological Surgery - Surgery C, The Chaim Sheba Medical Center, Tel Hashomer, Ramat Gan, Israel

**Keywords:** Carcinoid, Appendiceal mucinous neoplasm, Adenocarcinoid, Carcinogens, Cytoreductive surgery, HIPEC, Neuroendocrine tumor, Case series

## Abstract

**Background:**

Although uncommon, neoplasms of the appendix do exist. The two most common types are neuroendocrine tumors and mucinous appendiceal neoplasms.

**Methods:**

In two patients unusual gross and microscopic findings in an appendectomy specimen were recorded. Special immunocytochemistry studies were used to determine the histologic type of the tumors in the appendix.

**Results:**

The clinical features and histopathology of two patients who had both a neuroendocrine tumor and a low grade appendiceal mucinous neoplasm in the same appendectomy specimen were described. Possibilities for the causation and treatment of this unusual condition were discussed. The incidence of this double malignancy was estimated at 2.5 in 1000 appendectomy specimens.

**Conclusion:**

Although extremely unusual, a neuroendocrine tumor and a mucinous appendiceal neoplasm can exist in the same appendix. This condition is reported in two young patients.

## Highlights

• 0.5 % of appendectomy specimens show neuroendocrine and 0.5 % show epithelial malignancy.
• In 2 patients these two malignancies were in the same appendectomy specimen.
• Patients peritoneal metastases occurred from the mucinous epithelial malignancy only.
• Rather than routine right hemicolectomy, radical appendectomy is suggested.

## 1 Introduction

The most common pathology associated with the appendix is appendicitis. However, malignancy can occur. In a study of the Surveillance, Epidemiology and End-Results (SEER) database showed the annual incidence of primary appendiceal cancer was 0.12/1,000,000 [1]. A neuroendocrine (carcinoid) tumor is found in approximately 0.5 % of appendectomy specimens [2]. An epithelial appendiceal neoplasm occurs in approximately 0.5 % of appendectomy specimens [3]. Finding both these primary malignancies in the same appendectomy specimen must be an exceedingly rare event. Prior to this report a neuroendocrine tumor (carcinoid) and an epithelial neoplasm (mucinous neoplasm or adenocarcinoma) in the same appendectomy specimen has been reported only once [4]. We describe two malignancies of completely different histologies that were documented in the same appendectomy specimen.

## 2 Materials and methods

Data on these two patients was prospectively recorded and then retrospectively reviewed at academic institutions. This research work has been reported in line with the SCARE criteria [5]. This study was registered as a case report on the www.researchregistry.com website with UIN 5087.

## 3 Patient presentation

### 3.1 Patient 1

In June 2013, a 39-year-old woman noted right lower quadrant pain. This persisted and became localized in September of 2013 on the right side of the abdomen. A colonoscopy and upper GI endoscopy were negative except for H. pylori which was treated.

In October 2013, an ultrasound showed a mucocele of the appendix with septations. The diameter of the appendix was 2.3 cm and ascites was present in the right paracolic sulcus. The ovaries appeared normal and there was no fluid between liver and undersurface of the right hemidiaphragm.

On November 13, 2013, a diagnostic laparoscopy was performed and converted to an open right hemicolectomy. Extensive mucus was present within the right paracolic gutter and right side of the pelvis. Pathology report on the resected right colon showed a primary low grade appendiceal mucinous neoplasm which was ruptured with extrusion of mucin into the periappendiceal region. The surface of the small bowel showed foci of mucin and aggregates of epithelial cells consistent with a low grade mucinous neoplasm. The mucinous appendiceal neoplasm was considered to be at high risk for recurrence. Twenty-eight regional lymph nodes showed no evidence of metastatic carcinoma. Tumor stage for the mucinous appendiceal tumor was PT3N0M1a.

A second primary tumor was present within the appendiceal specimen. The was a “conventional neuroendocrine tumor, 1.7 cm in diameter”. The tumor stage for the neuroendocrine tumor was PT1BN0.

On April 10, 2015, the patient was again taken to the operating room for exploratory laparotomy. Mucin was present on the undersurface of the right hemidiaphragm, in and along the falciform ligament, and in the right paracolic sulcus. Procedures performed included a greater omentectomy, lesser omentectomy, cholecystectomy, hysterectomy and bilateral salpingo-oophorectomy [6]. Hyperthermic intraperitoneal chemotherapy (HIPEC) with mitomycin C and doxorubicin intraperitoneally and 5-fluorouracil and leucovorin systemically were administered for 90 min [7]. The chemotherapy solution was maintained at 42.5 °C.

Eleven separate specimens were submitted from the operating room to the pathologist. All specimens showed mucin aggregates and fibrosis. Pathology was thought to be consistent with disseminated peritoneal adenomucinosis with predominantly mucin escaped from the ruptured appendix into the free peritoneal cavity [8]. In December 2019, the patient remains well 5 years after her definitive cytoreduction with HIPEC.

### 3.2 Patient 2

A 32-year-old male patient underwent left inguinal hernia repair. During the surgery, the surgeon noticed mucin in the hernia sac which was submitted for histopathology. Histopathologic examination showed fibro-fatty tissue with accumulation of mucin. Patient underwent CT showing a "mucocele" of the appendix and fluid in the right paracolic gutter. Colonoscopy was then performed showing a tumor mass invading the cecum. Patient was referred to a peritoneal surface malignancy center.

On January 26, 2018 the patient underwent laparoscopy. Large mucin deposits were seen on the right hemidiaphragm, left upper quadrant and pelvis with a large tumor mass in the right lower quadrant of the abdomen. Peritoneal cancer index was 16 [9]. A decision was made to perform an open procedure.

Visceral resections were right colon, greater and lesser omentum, and gallbladder. Peritonectomy procedures were right subphrenic, left subphrenic, omental bursa, parietal and pelvic peritonectomies [7]. Several nodules were removed from the parietal surface of the distal jejunum and proximal ileum without bowel resection. The left groin mesh from the prior inguinal hernia repair was removed [10]. A complete cytoreduction (CC-0), was achieved. HIPEC was performed with mitomycin C for 90 min at inflow temperature of 44 °C and a mean abdominal temperature of 42 °C [7]. An additional outflow catheter was placed in the left groin. Postoperative course was unremarkable. The pathology report showed a primary low-grade mucinous neoplasm of the appendix [8]. The stage was PT3N0M1a. Acellular pools of mucin were seen in all 18 specimens submitted from the cytoreductive surgery.

A second primary well differentiated neuroendocrine tumor was present within the appendectomy specimen. Is showed Ki67 of 5 %. The neuroendocrine tumor was G2T2N1MX. Two of the 16 lymph nodes were involved by tumor. The patient underwent DOTATATE-Ga-68 scan showing no evidence of residual or metastatic disease. Chromogranin A and 5-HIAA were in the normal range postoperatively. The patient is being followed at 3-months intervals. He is fully active without evidence of disease recurrence in December of 2019.

### 3.3 Suggested incidence of two primary tumors in one appendix

If the incidence of carcinoid of the appendix is estimated at 0.5 % of appendectomy specimens and the incidence of a mucinous neoplasm of the appendix is 0.5 %. The incidence of both tumors in the same appendix may be estimated at 0.25 % or 2.5 in 1000 appendectomy specimens.

## 4 Discussion

### 4.1 Possible causation of the two different tumors in a single appendix

The incidence of appendiceal neoplasm is estimated at 1 % the incidence of colon and rectal cancer [3]. However, the surface area of the appendiceal mucosa is far less than 1 % of the surface area of the mucosa of the colon and rectum. It is possible that the appendix, which is a tubular structure with a blind end, has a prolonged exposure to retained intestinal carcinogens. The fact that neuroendocrine tumors and epithelial neoplasms, usually small and benign, both occur with a frequency of 0.5 % may also suggest increased levels of carcinogens in the appendix. It is possible that there is a carcinogen for neuroendocrine tumors of the appendix and another for adenomatous tumors. Now that both carcinoid and adenomatous tumors have been described in the same appendix specimen, it seems reasonable to speculate that the same carcinogen may be active in causation of both carcinoid and adenomatous appendiceal tumors.

### 4.2 Incidence of two different appendiceal tumors in an appendiceal malignancy database

A single specimen showing a double appendiceal neoplasm in Washington, DC occurred in a database of 1240 patients with epithelial appendiceal malignancies. However, since these patients were often referred after appendectomy that established the diagnosis of appendiceal neoplasm, this does not provide an incidence for a double neoplasm. It may be more common that is generally thought but pathologists have not found it necessary to establish the incidence of a double appendiceal neoplasm.

### 4.3 Treatment options for appendiceal malignancies

Neoplasm of the vermiform appendix are rare and therefore, treatment is mainly based on retrospective data, personal experience and extrapolation from colorectal cancer data. Since there are several types of appendiceal neoplasms, it is important to plan the treatment according to the underlying histology. The occurrence of two distinct primary tumors of different origin (neuroendocrine tumor and mucinous appendiceal neoplasm) is extremely rare. It was described once before in a case report [4]. Therefore, when coming to consider treatment options, one must extrapolate data and treatment guidelines from the individual appendiceal tumors.

### 4.4 Management of ileocolic lymph nodes

In neuroendocrine tumors of the appendix, larger than 20 mm, tumors invading the meso-appendix or tumors with lymphovascular invasion are at higher risk for lymph node metastasis. Therefore, right hemicolectomy has been recommended and is usually performed [11]. However, in this report by Moertel no survival advantage with right colon resection could be demonstrated. Also, in two recent reports, a search for improved survival with right hemicolectomy was not found [12,13]. A recent suggestion to help select patients for right colon resection is to perform a radical appendectomy with appendiceal and ileocolic lymph node sampling. Right hemicolectomy to resect occult positive lymph nodes was not shown to result in improved survival [14].

When diagnosis of both primary neuroendocrine tumor and primary mucinous appendiceal tumor is known before surgery, treatment can be planned. However, in many patients, the cross-sectional imaging shows the mucocele of the appendix but not the neuroendocrine tumor. This is revealed only in the final pathology. In case of low grade appendiceal neoplasm, there is a low risk for lymph node metastasis [15]. Appendectomy with clear margins combined with cytoreductive surgery and HIPEC in cases of rupture is sufficient. However, if an incidental neuroendocrine tumor is discovered in the surgical specimen, the question of re-operating and performing right hemicolectomy in order to clear lymph nodes at risk arises. Another strategy can be close follow up by DOTATATE-Ga-68 scan and serum Chromogranin-A, and intervene upon recurrence.

### 4.5 Contrast of the double primary tumor to goblet cell carcinoid

It is important to distinguish two primary tumors in the same appendectomy specimen from the combination of neuroendocrine tumor and mucin producing neoplasms of appendix in a single tumor known as goblet-cell carcinoid [16]. This tumor type has a poor prognosis, due to frequent peritoneal and lymphatic dissemination. Goblet-cell carcinoid is highly metastatic to lymph nodes and the peritoneum and as a result, CRS, including right hemicolectomy for lymph node clearance, combined with cytoreductive surgery and HIPEC are indicated for these patients.

However, similarities in the patterns of dissemination exist when the two primary appendiceal tumors are compared to the goblet cell carcinoid. In our two patients and the single patient reported by Hajjar et al., dissemination to peritoneal surfaces of the epithelial tumor was the metastatic disease [4]. The neuroendocrine tumor did not develop peritoneal metastases. A similar pattern of peritoneal dissemination of the epithelial component of goblet cell carcinoid to peritoneal surfaces was reported by Yan et al. [17]. The neuroendocrine tumor cells did not disseminate as peritoneal metastases in any of the 26 patients studied.

In summary, we describe two patients presenting with both a primary neuroendocrine tumor and a primary mucinous appendiceal tumor in the same appendectomy specimen successfully treated by cytoreductive surgery and HIPEC. It is extremely difficult to draw conclusions form two cases but with accumulating experience in high-volume centers, guidelines for treatment of these patients may be suggested. Until then, we recommend treatment of these patients with cytoreductive surgery and HIPEC in an experienced center.

## Sources of funding

Data management and secretarial support provided by Foundation for Applied Research in Gastrointestinal Oncology.

## Ethical approval

Local IRB-approval for this case report was not required:

MedStar Health Institutional Review Board has determined that a case report of less than three (3) patients **does not meet the DHHS definition of research** (45 CFR 46.102(d)(pre-2018)/45 CFR 46.102(l)(1/19/2017)) **or the FDA definition of clinical investigation** (21 CFR 46.102(c)) and therefore are not subject to IRB review requirements and **do not require IRB approval**.

This case report is of 2 patients.

## Consent

Written and signed consents were obtained from the patients.

## Author contribution

Paul H. Sugarbaker, MD: study concept or design, data collection, data analysis or interpretation, writing the paper

Almog Ben-Yaacov, MD: study concept or design, data collection, data analysis or interpretation, writing the paper

David Hazzan, MD: study concept or design, data collection, data analysis or interpretation, writing the paper

Aviram Nissan, MD: study concept or design, data collection, data analysis or interpretation, writing the paper

## Registration of research

This study was registered as a case report on the www.researchregistry.com website with UIN 5087.

## Guarantor

Paul H. Sugarbaker, MD

## Provenance and peer review

Not commissioned, externally peer-reviewed

## Declaration of Competing Interest

Paul H. Sugarbaker has no conflicts of interest to declare.
